# A statistical model to describe longitudinal and correlated metabolic risk factors: the Whitehall II prospective study

**DOI:** 10.1093/pubmed/fdv160

**Published:** 2015-11-06

**Authors:** P. Breeze, H. Squires, J. Chilcott, C. Stride, P.J. Diggle, E. Brunner, A. Tabak, A. Brennan

**Affiliations:** 1 School of Health and Related Research, University of Sheffield, Regent Court, 30 Regent Street, Sheffield S1 4DA, UK; 2 Institute of Work Psychology, University of Sheffield, Sheffield, UK; 3 Medical School, Lancaster University and Institute of Infection and Global Health, University of Liverpool, Liverpool, UK; 4 Epidemiology & Public Health, University College London, London, UK; 5 1st Department of Medicine, Semmelweis University Faculty of Medicine, Budapest, Hungary

**Keywords:** diabetes, epidemiology

## Abstract

**Background:**

Novel epidemiology models are required to link correlated variables over time, especially haemoglobin A1c (HbA1c) and body mass index (BMI) for diabetes prevention policy analysis. This article develops an epidemiology model to correlate metabolic risk factor trajectories.

**Method:**

BMI, fasting plasma glucose, 2-h glucose, HbA1c, systolic blood pressure, total cholesterol and high density lipoprotein (HDL) cholesterol were analysed over 16 years from 8150 participants of the Whitehall II prospective cohort study. Latent growth curve modelling was employed to simultaneously estimate trajectories for multiple metabolic risk factors allowing for variation between individuals. A simulation model compared simulated outcomes with the observed data.

**Results:**

The model identified that the change in BMI was associated with changes in glycaemia, total cholesterol and systolic blood pressure. The statistical analysis quantified associations among the longitudinal risk factor trajectories. Growth in latent glycaemia was positively correlated with systolic blood pressure and negatively correlated with HDL cholesterol. The goodness-of-fit analysis indicates reasonable fit to the data.

**Conclusions:**

This is the first statistical model that estimates trajectories of metabolic risk factors simultaneously for diabetes to predict joint correlated risk factor trajectories. This can inform comparisons of the effectiveness and cost-effectiveness of preventive interventions, which aim to modify metabolic risk factors.

## Introduction

There is growing interest in identifying effective and cost-effective interventions to prevent type 2 diabetes. There is evidence that public health interventions within the community are effective in improving healthy behaviours and reducing body mass index (BMI).^1–3^ In order to evaluate the cost-effectiveness of interventions, it is informative to describe progression to type 2 diabetes diagnosis in a simulation model. Therefore, it is useful to predict the longitudinal trajectory of glycaemia conditional on risk factors associated with diagnosis.

Previous policy analysis models have estimated progression to diabetes conditional on a single risk factor such as impaired glucose tolerance or BMI.^4,5^ It has been noted that other simulation models have simulated progression to diabetes independently of changes in other metabolic risk factors.^6^ Incorporating correlation between these factors is important in order to compare preventive interventions for three reasons. Firstly, multiple risk factors are used to identify individuals at high risk of diabetes. Secondly, interventions will affect multiple risk factors simultaneously. Thirdly, the relationship between these risk factors will affect the risk of other related conditions, such as cardiovascular disease.

Diabetes diagnosis is complicated because three tests can be used to assess an individual's glycaemic status. Thresholds for fasting and 2-h glucose and haemoglobin A1c (HbA1c) have been set for the diagnosis of type 2 diabetes.^7^ However, diabetes diagnosis and diabetes risk status may differ according to which test is used.^8,9^ A new predictive model for glycaemia trajectories should aim to describe the associations among glycaemic measures.

Previous analyses have estimated longitudinal trajectories for metabolic risk factors. Analyses of the Whitehall II cohort have investigated trajectories for metabolic risk factors in participants that progressed to diabetes diagnosis according to different diagnostic tests, and those remaining free from diabetes.^10^ The Baltimore Longitudinal Study of Aging has investigated the trajectory of the metabolic syndrome.^11^ In contrast, we aimed to develop a predictive model to describe trajectories for multiple risk factors within a single statistical analysis that captures interdependencies. Furthermore, in contrast with previous models, risk factors would be measured on a continuous scale, rather than dichotomized (e.g. hypertension and no-hypertension), to use all of the measurement information.

The aims of this study were to describe correlations and associations between changes in risk factors over time and predict the natural history of metabolic risk factors in a non-diabetic population.

## Methods

### Study data

Whitehall II is a longitudinal cohort study of UK civil servants. Phase 1 recruited 10 308 participants who worked in London and were aged 35–55 years between 1985 and 1988. The cohort was followed up in eight subsequent phases roughly 2.5 years apart. A questionnaire was administered in all phases, and every second phase included a clinical examination. In summary, 8815 attended Phase 3, 7870 attended Phase 5, 6967 attended Phase 7 and 6761 attended Phase 9. Participation details and baseline characteristics are provided in Supplementary data. The Whitehall II study was reviewed and approved by the University College London Ethics Committee (85/0938), and written informed consent was obtained at each phase. The study was conducted according to the principles of the Helsinki Declaration. Details of the cohort are described elsewhere.^12^

In Phases 3, 5, 7 and 9, observations were extracted from standard 2-h 75-g oral glucose tolerance tests (OGTTs), anthropometric measurements, blood pressure and total and high density lipoprotein (HDL) cholesterol. In Phases 7 and 9, HbA1c tests were available. Data on the participant's age, sex, ethnicity, smoking status at baseline, family history of diabetes and family history of cardiovascular disease were included in the study data set. Measures of socio-economic status were included in the analysis plan but were excluded because exploratory analysis indicated that socio-economic patterns observed from this historical cohort were not representative of forecasted patterns.

The OGTT was first taken in the Phase 3 clinical examination, so this was used as the baseline for our analysis.^13^ The study data set included all clinic visits attended up to Phase 9. We excluded 1075 (10.4%) participants who were lost to follow-up before Phase 3, 408 (4.0%) participants who did not contribute any clinical data in Phases 3, 5, 7 or 9, 136 (1.3%) participants with prevalent diabetes before Phase 3 and 439 (4.2%) participants with a history of cardiovascular disease or reported seeing a doctor for heart trouble. This left a final sample of 8150 participants (79.1% of the original sample).

At each study phase, criteria had been specified for blood glucose, blood pressure and cholesterol to alert the participant's general practitioner to elevated test results. It was, therefore, necessary to censor observations at this point where participation in the study may have altered the participant's metabolic risk factor trajectory.

### Latent growth curve modelling

The growth trajectory models for the metabolic risk factor were estimated under the statistical framework of latent growth curve modelling (LGCM).^14^ LGCM is an approach to using longitudinal data to estimate shape and rate of change over time. LGCM was chosen because it can allow modelling of both correlations within observations over time and variability between subjects, and enables the elegant modelling of change in multiple outcome variables. In LGCM, the baseline levels and the rates of change in the outcome(s) for each person are modelled as latent random variables, noisy ‘indicators’, which are measured at each time point. For example, if the hypothesized growth model is linear, the underlying latent variables we would seek to estimate are intercept and slope, respectively. The mean of the intercept describes the population-average baseline level and the mean of the slope the population-average rate of change. Additional slope factors can be added to the model for polynomial models. The indicators themselves can either be observed variables, or, if the construct can be measured by multiple tests, such as blood glucose levels, can be modelled as latent variables measured by a further set of observed ‘indicators’ in what is known as a second order, or curve of factors, LGCM.^15^ Once a basic LGCM is developed for each outcome, with means and variances estimated for intercept and slope factors, we can then extend the model. For example, we can explain between-subject variance in intercept and slope factors by adding time variant and invariant covariates, or where multiple LGCMs exist, by regressing the intercept and factors underlying one LGCM upon those of another LGCM.

### Conceptual model

**Fig. 1:**
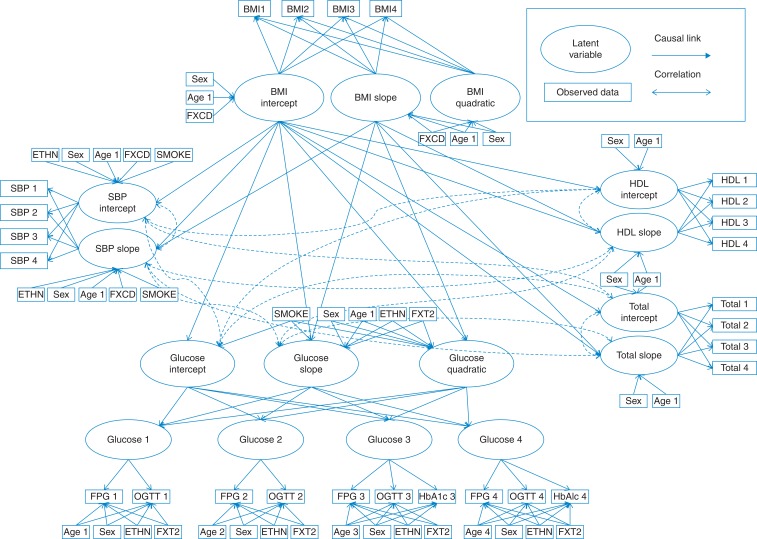
Path diagram for conceptual model.

We developed a conceptual model to describe the growth patterns for BMI, glycaemia, systolic blood pressure and total and HDL cholesterol over time with clinical experts. The conceptual model is illustrated in Fig. 1. The model assumed that BMI and glycaemia were quadratic to allow the rate of change to increase or decrease over time observed in other studies.^10,16^ The rate of BMI changes has been found to decrease in older age^16^ and glycaemia to increase prior to diagnosis.^10^ Glycaemia is described as a latent variable measured by fasting plasma glucose (FPG), and 2-h glucose in Phases 3 and 5 and FPG, 2-h glucose and HbA1c in Phases 7 and 9. It was hypothesized that change in systolic blood pressure total and HDL cholesterol was assumed to change linearly with time in line with observations from other studies.^10,17^

The conceptual model assumed that BMI intercept and linear slope growth factors were associated with the growth factors for glycaemia, systolic blood pressure, total cholesterol and HDL cholesterol. The BMI quadratic term describes the rate of deceleration in BMI growth due to ageing and was assumed to be unaffected by lifestyle factors that link BMI to other growth factors. The growth factors for glycaemia, systolic blood pressure and cholesterol were assumed correlated. Behavioural risk factors such as smoking, diet and physical activity were not included in the conceptual model to focus the conceptual model on reliable, externally valid outcomes that will be used in future cost-effectiveness models. Currently, the joint impact of behaviours and their impact on metabolic risk factors are not well understood and would add substantial complexity to the model.^18^

### Statistical analysis

The growth factors for the metabolic risk factors were assumed to vary between individuals to allow unobservable random effects to describe heterogeneity in the population. Correlation between the residual variance for growth factors for systolic blood pressure, glycaemia, total cholesterol and HDL cholesterol described correlation in their trajectories.

The LGCM for each metabolic risk factor was evaluated for goodness of fit separately before all were incorporated into the joint model along with the hypothesized covariates. We evaluated goodness of fit using the standardised root mean square residual (SRMR) cut-off criteria 0.08 and comparative fit index (CFI) cut-off criteria 0.95.^19^ The analyses were conducted using MPlusv7.11 software using full information maximum likelihood estimation. This will produce asymptotically unbiased estimates of means and standard errors assuming data are missing at random. We allowed the probability that a response is missing to depend arbitrarily on observed values of the response at other times, but not additionally on the unobserved response itself.^20^ We used sensitivity analyses to evaluate how robust the analyses were when missing observations were either excluded or imputed and found that the results did not change substantially.

A mathematical description of the model is presented in Supplementary data.

### Simulation study

A simulation model was developed to predict individual participant trajectories for the baseline characteristics of the Whitehall II participants from the parameters generated in the statistical analysis. We generated 100 sets of longitudinal trajectories of BMI, FPG, 2-h glucose, HbA1c, systolic blood pressure, total and HDL cholesterol conditional on the Whitehall II participant age, gender, ethnicity, smoking status and family history at 0, 6, 11 and 16 years of follow-up. The simulated observations at each phase of data were compared with the observed mean, variance and correlation. In addition, we plotted observations against age to assess whether the simulation reproduced age trends in the data. Finally, baseline characteristics and simulated metabolic data were used to generate risk scores for cardiovascular disease^21^ and diabetes^22^ at each time point using the observed and simulated data. These risk scores combine data from multiple metabolic risk factors to estimate the probability of long-term events.

## Results

The full list of parameters estimated from the statistical analysis is presented in Supplementary data, Tables S1–S3. Table 1A summarizes key model parameters describing the relationship between BMI and the other metabolic growth factors. The analysis identified that baseline BMI had a statistically significant effect on the baseline observations and growth rates for glycaemia, systolic blood pressure and total and HDL cholesterol. BMI growth rate had a statistically significant effect on growth rates for glycaemia, systolic blood pressure and total cholesterol. The effect of growth rate of BMI on the growth rate of HDL could not be identified. The results suggest that high BMI is associated with negative baseline values for the other risk factors. Increasing BMI over time is associated with higher growth rate for glycaemia, systolic blood pressure and total cholesterol. However, high baseline BMI had a negative effect on the growth rate of the other metabolic risk factors.

**Table 1:** Estimated parameters for relationship between BMI growth factors and other metabolic growth factors

*Dependent variable*	*Independent variable*	*Mean coefficient*	*Standard error*	**P*-value*
(A) BMI growth factor associations with other metabolic risk growth factors				
BMI intercept	Glycaemia intercept	0.2620	0.024	<0.001
	SBP intercept	0.1080	0.006	<0.001
	Total cholesterol intercept	0.4459	0.049	<0.001
	HDL cholesterol intercept	−0.3514	0.015	<0.001
	Glycaemia slope	0.0821	0.024	0.001
	SBP slope	−0.0396	0.006	<0.001
	Total cholesterol slope	−0.4808	0.035	<0.001
	HDL cholesterol slope	−0.0400	0.010	<0.001
BMI slope	Glycaemia slope	0.1984	0.073	0.007
	SBP slope	0.2325	0.019	<0.001
	Total cholesterol slope	0.9802	0.108	<0.001
(B) Covariate adjustments for FPG, 2-h glucose and HbA1c observations				
Age	FPG	0.0031	0.001	0.022
	2-h Glucose	0.0716	0.003	<0.001
	HbA1c	0.0101	0.001	<0.001
Sex (male = 1)	FPG	0.2129	0.021	<0.001
	2-h Glucose	−0.1411	0.058	0.014
	HbA1c	−0.0457	0.001	<0.001
Ethnicity (non-white = 1)	FPG	0.0100	0.037	0.786
	2-h Glucose	0.3047	0.100	0.002
	HbA1c	0.1854	0.030	<0.001
Family history of diabetes (family history = 1)	FPG	0.1168	0.025	<0.001
	2-h Glucose	0.3496	0.068	<0.001
	HbA1c	0.0563	0.020	0.004

The growth models of factors describe latent glycaemia, measured by FPG, 2-h glucose and HbA1c. HbA1c, FPG and 2-h glucose can be estimated at any time point according to a fixed population mean, plus a fixed linear association with latent glycaemia. The analysis identified differences in associations between FPG, 2-h glucose, HbA1c and individual characteristics (Table 1B). All measures were positively associated with age at phase of data, with 2-h glucose demonstrating the largest increase at older age. Males were found to report higher FPG tests, but lower 2-h glucose and HbA1c tests. Non-white ethnicity was not associated with FPG, but was associated with higher 2-h glucose and HBA1c observations. A family history of diabetes predicted higher scores for all glycaemic tests.

The goodness-of-fit statistics indicated a reasonable fit for a complex model. The SRMR test was in the region of the recommended threshold at 0.063 and the CFI slightly lower than the recommended threshold at 0.91. These fit statistics indicate that the model is a reasonable description of the data.

**Fig. 2:**
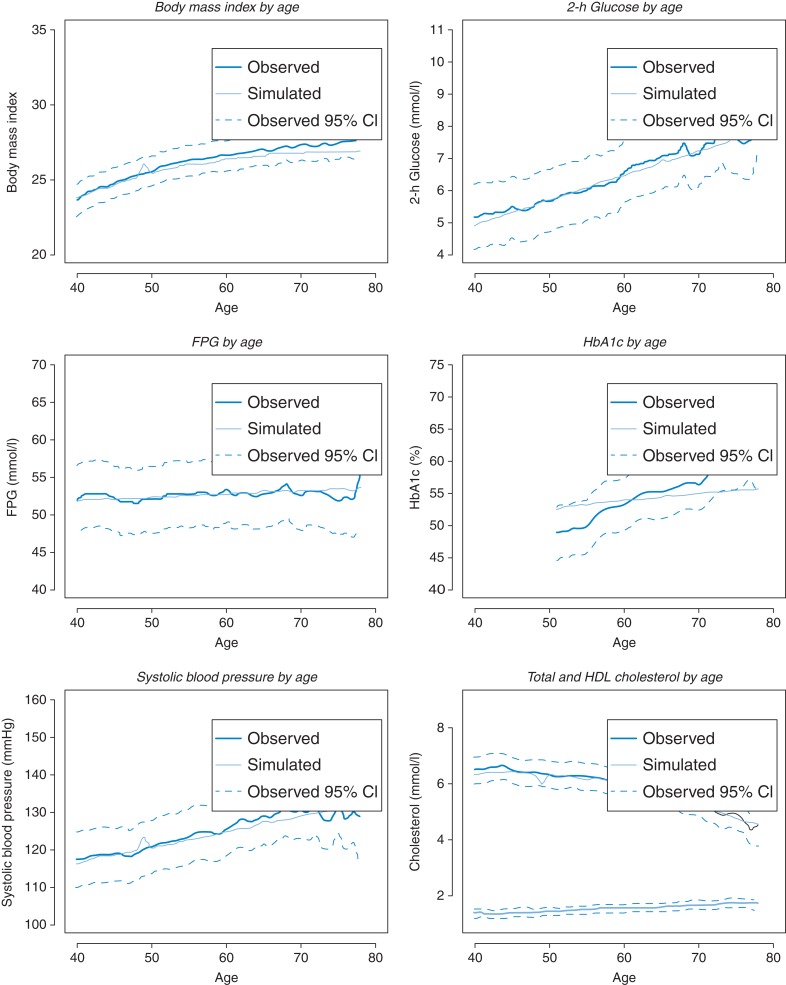
Observed and predicted expected metabolic risk scores by age.

The simulation study produced similar data to the original study data set. Average metabolic observations plotted against age are illustrated in Fig. 2. The simulated mean values were well within the 95% confidence intervals of the observed data for 2-h glucose, FPG, systolic blood pressure and total and HDL cholesterol. The simulation slightly underestimates BMI at older ages. The simulation does not reflect the steep trajectory for HbA1c by age observed in the data. However, it should be noted that these observations are based on less data than for the other metabolic risk factors. The lack of fit indicates that there may be problems simulating HbA1c trajectories with age and potentially indicates some structural inadequacy within the model. It is worth noting that the problem does not impact on the estimation of 2-h glucose, FPG or the correlations between these observations. Further validation is needed against an external data set to evaluate the reliability of HbA1c prediction. Illustrations of the distribution of simulated output compared with the data are illustrated in Supplementary data. The analysis suggests that the simulation predicts variability between individuals.

The second stage of the simulation aimed to assess correlation between metabolic risk factors. The correlation statistics at each phase of data for the study data set and simulated data are reported in the Supplementary data. The simulated correlations closely matched to the observed data. Table 2 reports the estimated Framingham risk score and diabetes risk scores at each phase of data for the observed and simulated data. The mean and standard deviations for the Framingham risk scores matched the observed data. The observed diabetes risk score increased in the first three phases of data and decreased in Phase 9, whereas the simulated risk score increased across all four phases. However, the means and standard deviations were similar. The average correlation statistics for participant simulated risk scores demonstrated positive association with the observed data and stronger association for cardiovascular risk than diabetes risk (Table 2).

**Table 2:** Observed and predicted Framingham risk score and diabetes risk score at each study phase for 100 simulations runs

	*Observed Framingham risk score (SD)*	*Simulated Framingham risk score (SD)*	*Difference*	*Mean correlation for participant observations and predicted values*
Phase 3	9.2% (6.6)	9.0% (4.5)	−0.18	0.761
Phase 5	11.2% (7.4)	11.4% (7.4)	0.19	0.717
Phase 7	13.3% (8.5)	12.7% (7.9)	−0.65	0.685
Phase 9	14.0% (8.4)	14.7% (8.9)	0.64	0.579
	*Observed diabetes score (SD)*	*Simulated diabetes score (SD)*	*Difference*	*Mean correlation for participant observations and predicted values*
Phase 3	19.4% (20.9)	18.4% (18.7)	−0.98	0.344
Phase 5	19.4% (20.4)	22.4% (21.5)	2.96	0.310
Phase 7	25.9% (26.2)	24.0% (23.5)	−1.93	0.319
Phase 9	22.5% (22.7)	27.6% (26.6)	5.14	0.300

SD, standard deviation.

## Discussion

### Main findings of this study

We have developed a statistical model to describe longitudinal trajectories in metabolic risk factors. By estimating growth trajectories simultaneously, it is possible to estimate dynamic associations between BMI and other risk factors, capture correlation between growth factors and heterogeneity in individuals' metabolic risks. The model can be used to extrapolate lifestyle changes and type 2 diabetes prevention strategies by predicting long-term changes in metabolic risk. The longitudinal trajectories for metabolic risk factors could be combined with epidemiology risk models for long-term health events, such as cardiovascular disease, cancer and mortality.^23,24^ Long-term cost savings and health benefits associated with reductions in these health events could be calculated by simulating changes to the longitudinal profile of metabolic risk factors. This would enable evaluation of alternative public health policies by estimating health and cost benefits.

### What is already known on this topic?

We have identified that growth in BMI is associated with increases in other metabolic risk factors over time, supporting previous findings that total fat and abdominal fat are associated with hyperglycaemia, hypertension and dyslipidaemia.^25–27^ The analysis identified that increases in BMI are associated with worsening in other metabolic risk factors, whereas the baseline BMI was weakly associated with increases in glycaemia and negatively associated with increases in systolic blood pressure and total cholesterol. A similar finding was observed when comparing baseline metabolic risk factors with those developed by diabetes diagnosis.^28^ High BMI at baseline is most likely associated with negative growth in systolic blood pressure and cholesterol due to an increased likelihood of high starting values for these measures initiating positive lifestyle changes.

### What this study adds

This study describes the first application of LGCM to metabolic risk factors in a good-quality longitudinal cohort to generate a natural history model for policy analysis simulations. This method enabled the simultaneous analysis of multiple growth trajectories in a single statistical analysis. The correlation between the longitudinal trajectories in this analysis was extremely important to accurately predict participants' future cardiovascular and diabetes risk, conditional on multiple metabolic risk factors. If the growth trajectories were assumed to be independent, the simulation would be more likely to under- or over-estimate these risks for an individual. This analysis was designed for use in a simulation to compare diabetes prevention interventions to allow policymakers to choose which interventions to fund. It was, therefore, important to consider the impact of modifying BMI trajectories on the longitudinal changes in glycaemia, systolic blood pressure and cholesterol to estimate reductions in the risk of diabetes and cardiovascular disease.

### Limitations

The data set, the choice of statistical framework and the software all imposed structural constraints on the statistical analysis. There is some variation in the time between clinical assessments for individuals within the data set, whereas assumed discrete time intervals in the model. We investigated alternative model specifications to allow for individually varying times of observation and to group observations by 5-year age ranges. However, these methods raised additional challenges; the model would not converge and the pairwise proportions of some variables present were zero in each case, respectively.

HbA1c was not available from the clinical assessments at Phases 3 and 5, which may explain why the fit to the data was worse for HbA1c. This results in an unbalanced measurement model for latent glycaemia between the early and later phases of observation. We attempted to approximate the missing observations using latent variables drawing on correlations with HbA1c observations from Phases 7 and 9. However, there was insufficient data to implement this analysis. Although the inclusion of HbA1c may cause some problems in the analysis, we believe the benefits for future simulation modelling justify its inclusion. HbA1c is an established diagnosis method for type 2 diabetes according to international and UK guidelines,^2,29^ is used for monitoring disease management and is a risk factor for diabetes complications.^30^ Our statistical analysis allows estimation of HbA1c from latent glycaemia, so that it is correlated with other blood glucose tests and also relates the test result to age and other participant characteristics. In the absence of a longitudinal cohort with all three glycaemic tests measured at regular intervals, this statistical analysis provides a best estimate of HbA1c conditional on multiple risk factors.

The simulation demonstrates that the analysis can fairly well reproduce the observed data from the Whitehall II study. However, the metabolic trajectories are not necessarily representative of the general population within the UK and other international settings. The Whitehall II data set is known to under-represent women and ethnic minorities in the UK.^12^ The longitudinal trajectories can be applied to alternative baseline characteristics to generate a more representative sample. Future research will aim to assess the external validity of the model in predicting the longitudinal trajectories from baseline.

## Supplementary data

Supplementary data are available here.

## Funding

This work was supported by the National Institute for Health Research School for Public Health Research (NIHR SPHR).

## Supplementary Material

Supplementary Data

## References

[1] PH35: Preventing Type 2 Diabetes: Population and Community-level Interventions. National Institute for Health and Care Excellence, 2011 NICE public health guidance 35 http://www.nice.org.uk/guidance/ph35 (25 October 2015, date last accessed).

[2] PH38 Preventing Type 2 Diabetes—Risk Identification and Interventions for Individuals at High Risk: Guidance. National Institute for Health and Care Excellence, 2012 NICE public health guidance 38 http://guidance.nice.org.uk/PH38/Guidance/pdf/English (25 October 2015, date last accessed).

[3] Tabak AG, Herder C, Rathmann W et al Prediabetes: a high-risk state for diabetes development. *Lancet* 2012;379 (9833):2279–90. 22683128 10.1016/S0140-6736(12)60283-9 PMC3891203

[4] Galani C, Schneider H, Rutten FF et al Modelling the lifetime costs and health effects of lifestyle intervention in the prevention and treatment of obesity in Switzerland. *Int J Public Health* 2007;52 (6):372–82. 18369000 10.1007/s00038-007-7014-9

[5] Gillies CL, Lambert PC, Abrams KR et al Different strategies for screening and prevention of type 2 diabetes in adults: cost effectiveness analysis. *BMJ* 2008;336 (7654):1180–5. 18426840 10.1136/bmj.39545.585289.25 PMC2394709

[6] Watson P, Preston L, Squires H et al Modelling the economics of type 2 diabetes mellitus prevention: a literature review of methods. *Appl Health Econ Health Policy* 2014;12 (3):239–53. 24595522 10.1007/s40258-014-0091-z

[7] World Health Organisation. Definition and Diagnosis of Diabetes Mellitus and Intermediate Hyperglycaemia. World Health Organisation, 2006 http://whqlibdoc.who.int/publications/2006/9241594934_eng.pdf (25 October 2015, date last accessed).

[8] Kernohan AF, Perry CG, Small M Clinical impact of the new criteria for the diagnosis of diabetes mellitus. *Clin Chem Lab Med* 2003;41 (9):1239–45. 14598876 10.1515/CCLM.2003.190

[9] Heianza Y, Hara S, Arase Y et al HbA1c 5.7–6.4% and impaired fasting plasma glucose for diagnosis of prediabetes and risk of progression to diabetes in Japan (TOPICS 3): a longitudinal cohort study. *Lancet* 2011;378 (9786):147–55. 21705064 10.1016/S0140-6736(11)60472-8

[10] Faerch K, Witte D, Tabak AG et al Trajectories of cardiometabolic risk factors before diagnosis of three subtypes of type 2 diabetes: a post-hoc analysis of the longitudinal Whitehall II cohort study. *Lancet Diab Endocrinol* 2013;1 (1):43–51. 10.1016/S2213-8587(13)70008-1 24622266

[11] Scuteri A, Morrell CH, Najjar SS et al Longitudinal paths to the metabolic syndrome: can the incidence of the metabolic syndrome be predicted? The Baltimore Longitudinal Study of Aging. *J Gerontol A Biol Sci Med Sci* 2009;64 (5):590–8. 19270183 10.1093/gerona/glp004 PMC4017826

[12] Marmot M, Brunner E Cohort profile: the Whitehall II study. *Int J Epidemiol* 2005;34 (2):251–6. 15576467 10.1093/ije/dyh372

[13] Brunner EJ, Marmot MG, Nanchahal K et al Social inequality in coronary risk: central obesity and the metabolic syndrome. Evidence from the Whitehall II study. *Diabetologia* 1997;40 (11):1341–9. 9389428 10.1007/s001250050830

[14] Duncan T, Duncan S *An Introduction to Latent Variable Growth Curve Modeling*. 2nd edn MahWah, NJ: Laurence Erlbaum, 2006.

[15] Preacher K, Wichman A, MacCallam RC et al *Latent Growth Curve Modelling*. London: Sage Publications, 2008.

[16] Arbeev KG, Ukraintseva SV, Akushevich I et al Age trajectories of physiological indices in relation to healthy life course. *Mech Ageing Dev* 2011;132 (3):93–102. 21262255 10.1016/j.mad.2011.01.001 PMC3064744

[17] Wills AK, Lawlor DA, Muniz-Terrera G et al Population heterogeneity in trajectories of midlife blood pressure. *Epidemiology* 2012;23 (2):203–11. 22249241 10.1097/EDE.0b013e3182456567 PMC3355297

[18] Scarborough P, Harrington RA, Mizdrak A et al The preventable risk integrated ModEl and its use to estimate the health impact of public health policy scenarios. *Scientifica (Cairo)* 2014;2014:748750. 25328757 10.1155/2014/748750 PMC4195430

[19] Hu L-T, Bentler P Cuttoff criteria for fit indexes in covariance structure analysis: conventional criteria versus new alternatives. *Struct Equation Model* 1999;6:1–55.

[20] Rubin DB Inference and missing data. *Biometrika* 1976;63 (3):581–92.

[21] D'Agostino RB Sr, Vasan RS, Pencina MJ et al General cardiovascular risk profile for use in primary care: the Framingham Heart Study. *Circulation* 2008;117 (6):743–53. 18212285 10.1161/CIRCULATIONAHA.107.699579

[22] Stern MP, Williams K, Haffner SM Identification of persons at high risk for type 2 diabetes mellitus: do we need the oral glucose tolerance test? *Ann Intern Med* 2002;136 (8):575–81. 11955025 10.7326/0003-4819-136-8-200204160-00006

[23] Clarke PM, Gray AM, Briggs A et al A model to estimate the lifetime health outcomes of patients with type 2 diabetes: the United Kingdom Prospective Diabetes Study (UKPDS) Outcomes Model (UKPDS no. 68). *Diabetologia* 2004;47 (10):1747–59. 15517152 10.1007/s00125-004-1527-z

[24] Hippisley-Cox J, Coupland C, Vinogradova Y et al Predicting cardiovascular risk in England and Wales: prospective derivation and validation of QRISK2. *BMJ* 2008;336 (7659):1475–82. 18573856 10.1136/bmj.39609.449676.25 PMC2440904

[25] Bot M, Spijkerman AM, Twisk JW et al Weight change over five-year periods and number of components of the metabolic syndrome in a Dutch cohort. *Eur J Epidemiol* 2010;25 (2):125–33. 20091093 10.1007/s10654-009-9419-7 PMC2821620

[26] Cameron AJ, Boyko EJ, Sicree RA et al Central obesity as a precursor to the metabolic syndrome in the AusDiab study and Mauritius. *Obesity (Silver Spring)* 2008;16 (12):2707–16. 18820650 10.1038/oby.2008.412

[27] Liu R, Brickman WJ, Christoffel KK et al Association of adiposity trajectories with insulin sensitivity and glycemic deterioration: a longitudinal study of rural Chinese twin adults. *Diabetes Care* 2012;35 (7):1506–12. 22596174 10.2337/dc11-2060 PMC3379613

[28] Gast GC, Spijkerman AM, Van der AD et al Five-year changes in biologic risk factors and risk of type 2 diabetes: are attained but not initial risk factor levels of importance? *Am J Epidemiol* 2012;176 (8):720–5. 23013621 10.1093/aje/kws189

[29] Use of Glycated Haemoglobin (HbA1c) in the Diagnosis of Diabetes Mellitus. World Health Organisation, 2011 http://www.who.int/diabetes/publications/report-hba1c_2011.pdf (25 October 2015, date last accessed).

[30] Hayes AJ, Leal J, Gray AM et al UKPDS outcomes model 2: a new version of a model to simulate lifetime health outcomes of patients with type 2 diabetes mellitus using data from the 30 year United Kingdom Prospective Diabetes Study: UKPDS 82. *Diabetologia* 2013;56 (9):1925–33. 23793713 10.1007/s00125-013-2940-y

