# Supplementary material for: A statistical model to describe longitudinal and correlated metabolic risk factors: the Whitehall II prospective study
Source: J Public Health (Oxf). 2015 Nov 6;38(4):679–87. doi: 10.1093/pubmed/fdv160 (PMC6092879; doi:10.1093/pubmed/fdv160)
Supplement: Supplementary Data [file fdv160_supplementary_data.zip › fdv160supp.docx]

Electronic Supplementary Material

Mathematical Equations

**BMI Latent Growth Curve Model**

BMI at any time period in the model is estimated using the following quadratic equation.

$${BMI}_{t}=\beta_{10}+\beta_{11}t+\beta_{12}t^{2}+\varepsilon_{1}$$

$$\beta_{10}=\alpha_{10}+\boldsymbol{\gamma}_{\boldsymbol{10}}\boldsymbol{X}+\upsilon_{10}$$

$$\beta_{11}=\alpha_{11}+\boldsymbol{\gamma}_{\boldsymbol{11}}\boldsymbol{X}+\upsilon_{11}$$

$$\beta_{12}=\alpha_{12}+\boldsymbol{\gamma}_{\boldsymbol{12}}\boldsymbol{X}$$

The intercept of the BMI calculation is described by $\beta_{10}$, the linear slope $\beta_{11}$ , quadratic term $\beta_{12}$ and a measurement error term $\varepsilon_{1}$. The intercept $\beta_{10}$ is conditional on the population mean intercept, $\alpha_{10}$, coefficients, $\boldsymbol{\gamma}_{\boldsymbol{10}}\boldsymbol{,}$for patient characteristics $\boldsymbol{X}$**,** and an individual level random error term $\upsilon_{10}$. Annual change in BMI is determined by $\beta_{11}$ and $\beta_{12}$, which are also conditional on population intercepts, covariate adjustments. The linear slope includes an individual patient random error term $\upsilon_{11}$, however the quadratic slope term does not.

**Latent Glycaemia Growth Curve Model**

Latent glycaemia (glyc) at any time period in the model is estimated using the following quadratic equation.

$${glyc}_{t}=\beta_{20}+\beta_{21}t+\beta_{22}t^{2}+\varepsilon_{2}$$

$$\beta_{20}=\alpha_{20}+\boldsymbol{\gamma}_{\boldsymbol{20}}\boldsymbol{X}+{\tau_{20}\beta_{10}+\upsilon}_{20}$$

$$\beta_{21}=\alpha_{21}+\boldsymbol{\gamma}_{\boldsymbol{21}}\boldsymbol{X+}\tau_{21}\beta_{10}+\tau_{22}\beta_{11}+\upsilon_{21}$$

$$\beta_{22}=\alpha_{22}+\boldsymbol{\gamma}_{\boldsymbol{22}}\boldsymbol{X}+\upsilon_{22}$$

The intercept of the latent glycaemia is described by $\beta_{20}$, the linear slope $\beta_{21}$ , quadratic term $\beta_{22}$ and a measurement error term $\varepsilon_{2}$. The intercept $\beta_{20}$ is conditional on the population mean intercept mean intercepts, $\alpha_{20}$, coefficients, $\boldsymbol{\gamma}_{\boldsymbol{20}}\boldsymbol{,}$for patient characteristics $\boldsymbol{X}$**,** the growth intercept for BMI $\beta_{10}$, and an individual level random error terms $\upsilon_{20}$. Annual change in latent glycaemia is determined by $\beta_{21}$ and $\beta_{22}$, which are also conditional on population intercepts, covariate adjustments and individual level random error terms. The growth factors $\beta_{20}$and $\beta_{21}$ are also conditional on the growth factors for BMI.

The glycaemic test results (FPG, 2Hr Glucose, HbA1c) were assumed to be conditional on latent glycaemia, *glyc*. The model estimates test results for each period of observation (*t=*1,2,3,4…*t*). The factor glycaemia is measured by three non-overlapping observations of 2-hr, FPG and HbA1c. The scale of the factor is fixed by setting one factor loading (FPG) to 1. The tests were also conditional on age, sex, ethnicity and a family history of diabetes.

$$\left[ \begin{matrix} {FPG}_{t} \\ {2HR}_{t} \\ {A1C}_{t} \end{matrix} \right]=\left[ \begin{matrix} \mu_{0} \\ \mu_{1} \\ \mu_{2} \end{matrix} \right]+\left[ \begin{matrix} 1 \\ \theta_{11} \\ \theta_{21} \end{matrix} \right]\left[ {glyc}_{t} \right]+\left[ \begin{matrix} \theta_{02} \\ \theta_{12} \\ \theta_{22} \end{matrix} \right]\left[ {AGE}_{t} \right]+\left[ \begin{matrix} \theta_{03} \\ \theta_{13} \\ \theta_{23} \end{matrix} \right]\left[ SEX \right]+\left[ \begin{matrix} \theta_{04} \\ \theta_{14} \\ \theta_{24} \end{matrix} \right]\left[ ETHN \right]+\left[ \begin{matrix} \theta_{05} \\ \theta_{15} \\ \theta_{25} \end{matrix} \right]\left[ FXT2 \right]+\left[ \begin{matrix} \varepsilon_{20} \\ \varepsilon_{21} \\ \varepsilon_{22} \end{matrix} \right]$$

**Systolic Blood Pressure Growth Curve Model**

Systolic blood Pressure (SBP) at any time period in the model is estimated using the following linear equation.

$${SBP}_{t}=\beta_{30}+\beta_{31}t+\varepsilon_{3}$$

$$\beta_{30}=\alpha_{30}+\boldsymbol{\gamma}_{\boldsymbol{30}}\boldsymbol{X}+{\tau_{30}\beta_{10}+\upsilon}_{30}$$

$$\beta_{31}=\alpha_{31}+\boldsymbol{\gamma}_{\boldsymbol{31}}\boldsymbol{X+}\tau_{31}\beta_{10}\boldsymbol{+}\tau_{32}\beta_{11}+\upsilon_{31}$$

The intercept of the systolic blood pressure growth model is described by $\beta_{30}$, the linear slope $\beta_{31}$ and a measurement error term $\varepsilon_{3}$. The intercept $\beta_{30}$ is conditional on the population mean intercept mean intercepts, $\alpha_{30}$, coefficients, $\boldsymbol{\gamma}_{\boldsymbol{30}}\boldsymbol{,}$for patient characteristics $\boldsymbol{X}$**,** the growth intercept for BMI, and an individual level random error terms $\upsilon_{30}$. Annual change in SBP is determined by $\beta_{31}$, which is also conditional on population intercepts, covariate adjustments and an individual level random error term. Growth in SBP is also conditional on baseline BMI and the growth rate of BMI.

**Total Cholesterol Growth Curve Model**

Total Cholesterol (TC) at any time period in the model is estimated using the following linear equation.

$${TC}_{t}=\beta_{40}+\beta_{41}t+\varepsilon_{4}$$

$$\beta_{40}=\alpha_{40}+\boldsymbol{\gamma}_{\boldsymbol{40}}\boldsymbol{X}+{\tau_{40}\beta_{10}+\upsilon}_{40}$$

$$\beta_{41}=\alpha_{41}+\boldsymbol{\gamma}_{\boldsymbol{41}}\boldsymbol{X+}\tau_{41}\beta_{10}\boldsymbol{+}\tau_{41}\beta_{11}+\upsilon_{41}$$

The intercept of the systolic blood pressure growth model is described by $\beta_{40}$, the linear slope $\beta_{41}$ and a measurement error term $\varepsilon_{4}$. The intercept $\beta_{40}$ is conditional on the population mean intercept mean intercepts, $\alpha_{40}$, coefficients, $\boldsymbol{\gamma}_{\boldsymbol{40}}\boldsymbol{,}$for patient characteristics $\boldsymbol{X}$**,** a factor, $\tau_{40}$, describing the association with the growth intercept for BMI $\beta_{10}$, and an individual level random error terms $\upsilon_{40}$. Annual change in TC is determined by $\beta_{41}$, which is also conditional on population intercepts, covariate adjustments and an individual level random error term. Growth in TC is conditional on baseline BMI and the growth rate of BMI.

**HDL Cholesterol Growth Curve Model**

HDL Cholesterol (HDL) at any time period in the model is estimated using the following linear equation.

$${HDL}_{t}=\beta_{50}+\beta_{51}t+\varepsilon_{5}$$

$$\beta_{50}=\alpha_{50}+\boldsymbol{\gamma}_{\boldsymbol{50}}\boldsymbol{X}+{\tau_{51}\beta_{10}+\upsilon}_{50}$$

$$\beta_{51}=\alpha_{51}+\boldsymbol{\gamma}_{\boldsymbol{51}}\boldsymbol{X+}\tau_{51}\beta_{10}+\upsilon_{51}$$

The intercept of the systolic blood pressure growth model is described by $\beta_{50}$, the linear slope $\beta_{51}$ and a measurement error term $\varepsilon_{5}$. The intercept $\beta_{50}$ is conditional on the population mean intercept mean intercepts, $\alpha_{50}$, coefficients, $\boldsymbol{\gamma}_{\boldsymbol{50}}\boldsymbol{,}$for patient characteristics $\boldsymbol{X}$**,** a factor, $\tau_{50}$, describing the association with the growth intercept for BMI $\beta_{10}$, and an individual level random error terms $\upsilon_{50}$. Annual change in HDL is determined by $\beta_{51}$, which is also conditional on population intercepts, covariate adjustments and an individual level random error term. Growth in HDL is conditional on baseline BMI only.

Statistical Model Parameters

Table i: Coefficient estimates for metabolic risk factor parallel growth models

|  | Parameter Description | Estimated Mean | Standard error | p-value |
| --- | --- | --- | --- | --- |
| BMI Intercept | |  |  |  |
| $\alpha_{10}$ | Population mean BMI intercept | 2.2521 | 0.045 | <0.001 |
| $\boldsymbol{\gamma}_{\boldsymbol{10}}$ | Age at baseline coefficient for BMI intercept | 0.0056 | 0.001 | <0.001 |
|  | Sex coefficient for BMI intercept | -0.0311 | 0.012 | 0.009 |
|  | Family history of CVD coefficient for BMI intercept | -0.0079 | 0.012 | 0.515 |
| $\upsilon_{10}$ | Random error term for BMI intercept | 0.1165 | 0.003 | <0.001 |
| BMI linear slope | |  |  |  |
| $\alpha_{11}$ | Population mean BMI linear slope | 0.6409 | 0.042 | <0.001 |
| $\boldsymbol{\gamma}_{\boldsymbol{11}}$ | Age at baseline coefficient for BMI linear slope | -0.0084 | 0.001 | <0.001 |
|  | Sex coefficient for BMI linear slope | -0.0285 | 0.011 | 0.009 |
|  | Family history of CVD coefficient for BMI linear slope | -0.0155 | 0.010 | 0.117 |
| $\upsilon_{11}$ | Random error term for BMI linear slope | 0.0222 | <0.001 | <0.001 |
| BMI quadratic slope | |  |  |  |
| $\alpha_{12}$ | Population mean BMI quadratic slope | -0.2007 | 0.023 | <0.001 |
| $\boldsymbol{\gamma}_{\boldsymbol{12}}$ | Age at baseline coefficient for quadratic slope | 0.0026 | <0.001 | <0.001 |
|  | Sex coefficient for quadratic slope | 0.0089 | 0.006 | 0.147 |
|  | Family history of CVD coefficient for quadratic slope | 0.0104 | 0.006 | 0.061 |
| $\varepsilon_{1}$ | Random error term for BMI | 0.0104 | <0.001 | <0.001 |
| Glyc Intercept | |  |  |  |
| $\alpha_{20}$ | Population mean glyc intercept | 0 | NA | NA |
| $\boldsymbol{\gamma}_{\boldsymbol{20}}$ | Smoker coefficient for glyc intercept | -0.1388 | 0.029 | <0.001 |
| $\tau_{20}$ | Association between BMI intercept and glyc intercept | 0.2620 | 0.024 | <0.001 |
| $\upsilon_{20}$ | Random error term for glyc intercept | 0.0851 | 0.008 | <0.001 |
| Glyc linear slope | |  |  |  |
| $\alpha_{21}$ | Population mean glyc linear slope | -0.4255 | 0.071 | <0.001 |
| $\boldsymbol{\gamma}_{\boldsymbol{21}}$ | Sex coefficient for glyc linear slope | 0.1486 | 0.045 | 0.001 |
|  | Ethnicity coefficient for glyc linear slope | -0.0218 | 0.081 | 0.786 |
|  | Family history of T2DM coefficient for glyc linear slope | -0.0512 | 0.054 | 0.345 |
|  | Smoker coefficient for glyc linear slope | 0.1796 | 0.066 | 0.007 |
| $\tau_{21}$ | Association between BMI intercept and glyc linear slope | 0.0821 | 0.024 | 0.001 |
| $\tau_{22}$ | Association between BMI linear slope and glyc linear slope | 0.1984 | 0.073 | 0.007 |
| $\upsilon_{21}$ | Random error term for glyc linear slope | 0.0222 | 0.011 | 0.053 |
| Glyc quadratic slope | |  |  |  |
| $\alpha_{22}$ | Population mean glyc quadratic slope | 0.1094 | 0.025 | <0.001 |
| $\boldsymbol{\gamma}_{\boldsymbol{22}}$ | Sex coefficient for glyc quadratic slope | -0.0855 | 0.027 | 0.002 |
|  | Ethnicity coefficient for glyc quadratic slope | 0.0899 | 0.049 | 0.067 |
|  | Family history of T2DM coefficient for glyc quadratic slope | 0.0633 | 0.033 | 0.052 |
|  | Smoker coefficient for glyc quadratic slope | -0.0390 | 0.040 | 0.330 |
| $\upsilon_{22}$ | Random error term for glyc quadratic slope | 0.0107 | 0.003 | 0.002 |
| $\varepsilon_{2}$ | Glyc measurement error | 0.0707 | 0.005 | <0.001 |
| SBP Intercept | |  |  |  |
| $\alpha_{30}$ | Population mean SBP intercept | 0.6934 | 0.021 | <0.001 |
| $\boldsymbol{\gamma}_{\boldsymbol{30}}$ | Age at baseline coefficient for SBP intercept | 0.0043 | <0.001 | <0.001 |
|  | Sex coefficient for SBP intercept | 0.0380 | 0.004 | <0.001 |
|  | Smoking coefficient for SBP intercept | -0.0243 | 0.006 | <0.001 |
|  | Ethnicity coefficient for SBP intercept | 0.0078 | 0.007 | 0.300 |
|  | Family history of CVD coefficient for SBP intercept | 0.0061 | 0.004 | 0.160 |
| $\boldsymbol{\tau}_{\boldsymbol{31}}$ | Association between BMI intercept and SBP intercept | 0.1080 | 0.006 | <0.001 |
| $\upsilon_{30}$ | Random error term for SBP intercept | 0.0085 | 0.00 | <0.001 |
| SBP linear slope | |  |  |  |
| $\alpha_{31}$ | Population mean SBP linear slope | -0.0227 | 0.021 | 0.278 |
| $\boldsymbol{\gamma}_{\boldsymbol{31}}$ | Age at baseline coefficient for SBP linear slope | 0.0024 | <0.001 | <0.001 |
|  | Sex coefficient for SBP linear slope | -0.0004 | 0.004 | 0.927 |
|  | Smoking coefficient for SBP linear slope | 0.0205 | 0.005 | <0.001 |
|  | Ethnicity coefficient for SBP linear slope | 0.0224 | 0.007 | 0.001 |
|  | Family history of CVD coefficient for SBP linear slope | -0.0013 | 0.004 | 0.748 |
| $\boldsymbol{\tau}_{\boldsymbol{31}}$ | Association between BMI intercept and SBP linear slope | -0.0396 | 0.006 | <0.001 |
|  | Association between BMI linear slope and SBP linear slope | 0.2325 | 0.019 | <0.001 |
| $\upsilon_{31}$ | Random error term for SBP linear slope | 0.0024 | <0.001 | <0.001 |
| $\varepsilon_{3}$ | SBP measurement error variance | 0.0093 | <0.001 | <0.001 |
| TC Intercept | |  |  |  |
| $\alpha_{40}$ | Population mean TC intercept | 2.9956 | 0.176 | <0.001 |
| $\boldsymbol{\gamma}_{\boldsymbol{40}}$ | Age at baseline coefficient for TC intercept | 0.0456 | 0.003 | <0.001 |
|  | Sex coefficient for TC intercept | 0.0660 | 0.036 | 0.070 |
| $\tau_{40}$ | Association between BMI intercept and TC intercept | 0.4459 | 0.049 | <0.001 |
| $\upsilon_{40}$ | Random error term for TC intercept | 0.8960 | 0.025 | <0.001 |
| TC linear slope | |  |  |  |
| $\alpha_{41}$ | Population mean TC linear slope | 2.1216 | 0.128 | <0.001 |
| $\boldsymbol{\gamma}_{\boldsymbol{41}}$ | Age at baseline coefficient for TC linear slope | -0.0316 | 0.002 | <0.001 |
|  | Sex coefficient for TC linear slope | -0.2677 | 0.026 | <0.001 |
| $\tau_{41}$ | Association between BMI intercept and TC linear slope | -0.4808 | 0.035 | <0.001 |
| $\tau_{42}$ | Association between BMI linear slope and TC linear slope | 0.9802 | 0.108 | <0.001 |
| $\upsilon_{41}$ | Random error term for TC linear slope | 0.1583 | 0.011 | <0.001 |
| $\varepsilon_{4}$ | TC measurement error variance | 0.3426 | 0.006 | <0.001 |
| HDL Intercept | |  |  |  |
| $\alpha_{50}$ | Population mean HDL intercept | 2.4124 | 0.054 | <0.001 |
| $\boldsymbol{\gamma}_{\boldsymbol{50}}$ | Age at baseline coefficient for HDL intercept | 0.0032 | 0.011 | <0.001 |
|  | Sex coefficient for HDL intercept | -0.3710 | 0.001 | <0.001 |
| $\tau_{51}$ | Association between BMI intercept and HDL intercept | -0.3514 | 0.015 | <0.001 |
| $\upsilon_{50}$ | Random error term for HDL intercept | 0.0827 | -0.040 | <0.001 |
| HDL linear slope | |  |  |  |
| $\alpha_{51}$ | Population mean HDL linear slope | 0.1241 | 0.034 | <0.001 |
| $\boldsymbol{\gamma}_{\boldsymbol{51}}$ | Age at baseline coefficient for HDL linear slope | 0.0020 | 0.001 | <0.001 |
|  | Sex coefficient for HDL linear slope | 0.0041 | 0.007 | 0.558 |
| $\boldsymbol{\tau}_{\boldsymbol{51}}$ | Association between BMI intercept and HDL linear slope | -0.0400 | 0.010 | <0.001 |
| $\upsilon_{51}$ | Random error term for HDL linear slope | 0.0090 | 0.001 | <0.001 |
| $\varepsilon_{5}$ | HDL measurement error variance | 0.0333 | 0.001 | <0.001 |

Table ii: Coefficient estimates for latent glycaemic measurement model

|  | Parameter Description | Estimated Mean | Standard error | p-value |
| --- | --- | --- | --- | --- |
| $\mu_{0}$ | FPG intercept | 4.2903 | 0.089 | <0.001 |
| $\theta_{01}$ | Glycaemic factor to FPG | 1 | NA | NA |
| $\theta_{02}$ | Age to FPG | 0.0031 | 0.001 | 0.022 |
| $\theta_{03}$ | Sex to FPG | 0.2129 | 0.021 | <0.001 |
| $\theta_{04}$ | Ethnicity to FPG | 0.0100 | 0.037 | 0.786 |
| $\theta_{05}$ | Family history of diabetes to FPG | 0.1168 | 0.025 | <0.001 |
| $\varepsilon_{0}$ | FPG measurement error variance | 0.1649 | 0.007 | <0.001 |
| $\mu_{1}$ | 2-hr Glucose intercept | 0.5707 | 0.223 | 0.011 |
| $\theta_{11}$ | Glycaemic factor to 2-hr glucose | 2.4384 | 0.078 | <0.001 |
| $\theta_{12}$ | Age to 2-hr glucose | 0.0716 | 0.003 | <0.001 |
| $\theta_{13}$ | Sex to 2-hr glucose | -0.1411 | 0.058 | 0.014 |
| $\theta_{14}$ | Ethnicity to 2-hr glucose | 0.3047 | 0.100 | 0.002 |
| $\theta_{15}$ | Family history of diabetes to 2-hr glucose | 0.3496 | 0.068 | <0.001 |
| $\varepsilon_{1}$ | 2-hr measurement error variance | 2.3679 | 0.054 | <0.001 |
| $\mu_{2}$ | HbA1c intercept | 4.4769 | 0.073 | <0.001 |
| $\theta_{21}$ | Glycaemic factor to HBA1c | 0.5074 | 0.016 | <0.001 |
| $\theta_{22}$ | Age to HBA1c | 0.0101 | 0.001 | <0.001 |
| $\theta_{23}$ | Sex to HBA1c | -0.0457 | 0.001 | <0.001 |
| $\theta_{24}$ | Ethnicity to HBA1c | 0.1854 | 0.030 | <0.001 |
| $\theta_{25}$ | Family history of diabetes to HBA1c | 0.0563 | 0.020 | 0.004 |
| $\varepsilon_{2}$ | HbA1c measurement error variance | 0.1166 | 0.003 | <0.001 |

Table iii: Covariance matrix $\boldsymbol{\Omega}$ for individual random error

|  | $\upsilon_{10}$ | $\upsilon_{11}$ | $\upsilon_{20}$ | $\upsilon_{21}$ | $\upsilon_{22}$ | $\upsilon_{30}$ | $\upsilon_{31}$ | $\upsilon_{40}$ | $\upsilon_{41}$ | $\upsilon_{50}$ | $\upsilon_{51}$ |
| --- | --- | --- | --- | --- | --- | --- | --- | --- | --- | --- | --- |
| $\upsilon_{10}$ | 0.1165 |  |  |  |  |  |  |  |  |  |  |
| $\upsilon_{11}$ | 0.0095 | 0.0131 |  |  |  |  |  |  |  |  |  |
| $\upsilon_{20}$ | <0.0010 | <0.0010 | 0.0851 |  |  |  |  |  |  |  |  |
| $\upsilon_{21}$ | <0.0010 | <0.0010 | 0.0222 | 0.0209 |  |  |  |  |  |  |  |
| $\upsilon_{22}$ | <0.0010 | <0.0010 | <0.0010 | <0.0010 | 0.0107 |  |  |  |  |  |  |
| $\upsilon_{30}$ | <0.0010 | <0.0010 | 0.0080 | <0.0010 | <0.0010 | 0.0085 |  |  |  |  |  |
| $\upsilon_{31}$ | <0.0010 | <0.0010 | <0.0010 | 0.0018 | <0.0010 | <0.0017 | 0.0024 |  |  |  |  |
| $\upsilon_{40}$ | <0.0010 | <0.0010 | 0.0324 | <0.0010 | <0.0010 | 0.0031 | <0.0010 | 0.8960 |  |  |  |
| $\upsilon_{41}$ | <0.0010 | <0.0010 | <0.0010 | -<0.0012 | <0.0010 | <0.0010 | 0.0066 | -0.2229 | 0.1583 |  |  |
| $\upsilon_{50}$ | <0.0010 | <0.0010 | -0.0118 | <0.0010 | <0.0010 | 0.0010 | <0.0010 | 0.0273 | <0.0010 | 0.0827 |  |
| $\upsilon_{51}$ | <0.0010 | <0.0010 | <0.0010 | -0.0059 | <0.0010 | <0.0010 | 0.0020 | <0.0010 | 0.0159 | 0.0061 | 0.0090 |

Evaluation of model fit

Figure i: Histogram of Whitehall II BMI observations by Phase and simulated density


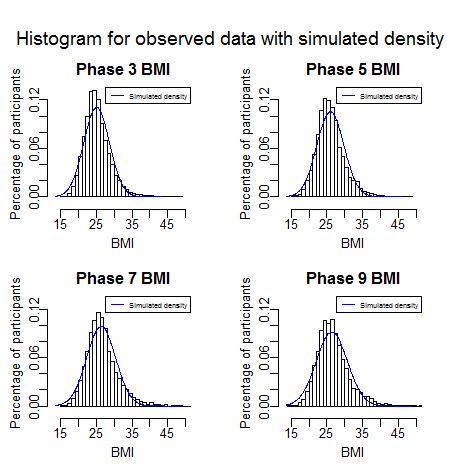


Figure ii: Histogram of Whitehall II 2hr glucose observations by Phase and simulated density


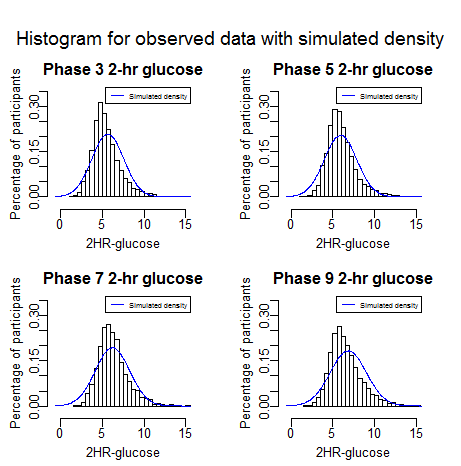


Figure iii: Histogram of Whitehall II FPG observations by Phase and simulated density


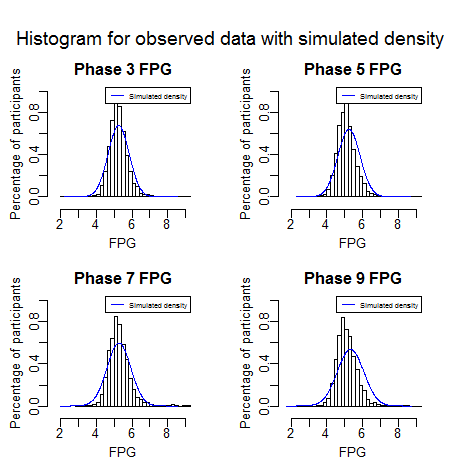


Figure iv: Histogram of Whitehall II HbA1c observations by Phase and simulated density

* HbA1c was not collected in Phases 3 and 5. The density plot illustrates the simulated values.

Figure v: Histogram of Whitehall II systolic blood pressure observations by Phase and simulated density


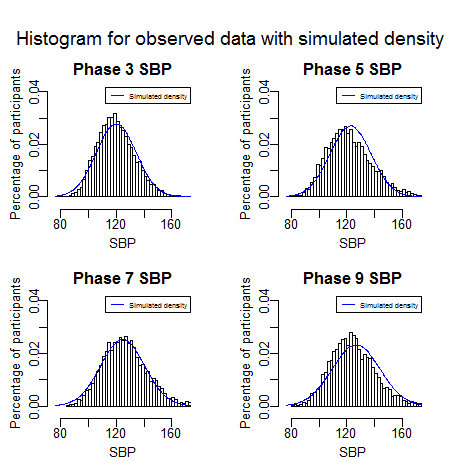


Figure vi: Histogram of Whitehall II total cholesterol observations by Phase and simulated density


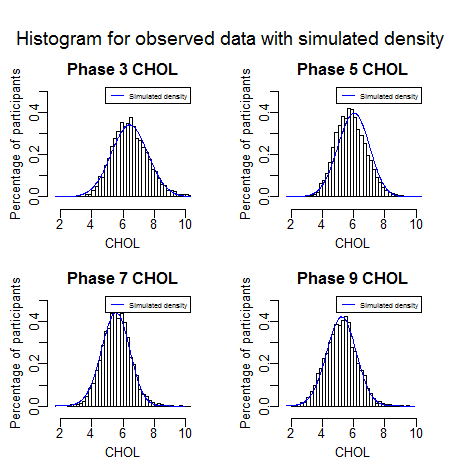


Figure vii: Histogram of Whitehall II HDL cholesterol observations by Phase and simulated density


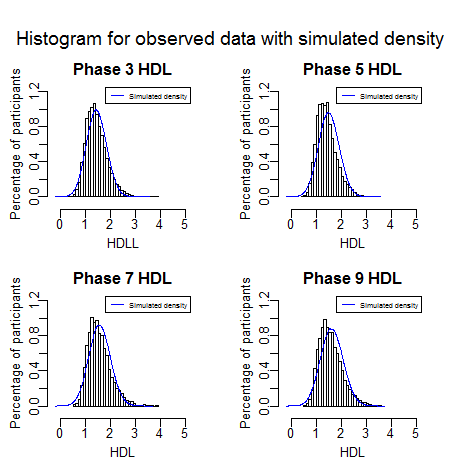


Correlation Matrix

Table iv: Correlation matrix Phase 3

|  | Real Whitehall Data: Phase 3 | | | | | | |  | Simulated Data: Phase 3 | | | | | | |
| --- | --- | --- | --- | --- | --- | --- | --- | --- | --- | --- | --- | --- | --- | --- | --- |
|  | BMI | G2HR | FPG | HBA | SBP | CHOL | HDL |  | BMI | G2HR | FPG | HBA | SBP | CHOL | HDL |
| BMI | 1.000 |  |  |  |  |  |  | BMI | 1.000 |  |  |  |  |  |  |
| G2HR | 0.144 | 1.000 |  |  |  |  |  | G2HR | 0.135 | 1.000 |  |  |  |  |  |
| FPG | 0.195 | 0.570 | 1.000 |  |  |  |  | FPG | 0.144 | 0.371 | 1.000 |  |  |  |  |
| HBA |  |  |  | 1.000 |  |  |  | HBA | 0.125 | 0.317 | 0.350 | 1.000 |  |  |  |
| SBP | 0.251 | 0.165 | 0.235 |  | 1.000 |  |  | SBP | 0.262 | 0.149 | 0.235 | 0.235 | 1.000 |  |  |
| CHOL | 0.147 | 0.067 | 0.079 |  | 0.135 | 1.000 |  | CHOL | 0.147 | 0.109 | 0.108 | 0.079 | 0.135 | 1.000 |  |
| HDL | -0.262 | -0.086 | -0.124 |  | -0.091 | 0.060 | 1.000 | HDL | -0.266 | -0.041 | -0.124 | -0.124 | -0.091 | 0.060 | 1.000 |

Table v: Correlation matrix Phase 5

|  | Real Whitehall Data: Phase 5 | | | | | | |  | Simulated Data: Phase 5 | | | | | | |
| --- | --- | --- | --- | --- | --- | --- | --- | --- | --- | --- | --- | --- | --- | --- | --- |
|  | BMI | G2HR | FPG | HBA | SBP | CHOL | HDL |  | BMI | G2HR | FPG | HBA | SBP | CHOL | HDL |
| BMI | 1.000 |  |  |  |  |  |  | BMI | 1.000 |  |  |  |  |  |  |
| G2HR | 0.152 | 1.000 |  |  |  |  |  | G2HR | 0.135 | 1.000 |  |  |  |  |  |
| FPG | 0.172 | 0.385 | 1.000 |  |  |  |  | FPG | 0.160 | 0.420 | 1.000 |  |  |  |  |
| HBA |  |  |  | 1.000 |  |  |  | HBA | 0.131 | 0.349 | 0.398 | 1.000 |  |  |  |
| SBP | 0.271 | 0.187 | 0.153 |  | 1.000 |  |  | SBP | 0.226 | 0.139 | 0.154 | 0.120 | 1.000 |  |  |
| CHOL | 0.127 | 0.058 | 0.074 |  | 0.130 | 1.000 |  | CHOL | 0.073 | 0.064 | 0.050 | 0.047 | 0.073 | 1.000 |  |
| HDL | -0.265 | -0.077 | -0.114 |  | -0.027 | 0.090 | 1.000 | HDL | -0.271 | -0.075 | -0.187 | -0.055 | -0.065 | 0.088 | 1.000 |

Table vi: Correlation matrix Phase 7

|  | Real Whitehall Data: Phase 7 | | | | | | |  | Simulated Data: Phase 7 | | | | | | |
| --- | --- | --- | --- | --- | --- | --- | --- | --- | --- | --- | --- | --- | --- | --- | --- |
|  | BMI | G2HR | FPG | HBA | SBP | CHOL | HDL |  | BMI | G2HR | FPG | HBA | SBP | CHOL | HDL |
| BMI | 1.000 |  |  |  |  |  |  | BMI | 1.000 |  |  |  |  |  |  |
| G2HR | 0.163 | 1.000 |  |  |  |  |  | G2HR | 0.145 | 1.000 |  |  |  |  |  |
| FPG | 0.231 | 0.469 | 1.000 |  |  |  |  | FPG | 0.173 | 0.479 | 1.000 |  |  |  |  |
| HBA | 0.212 | 0.394 | 0.540 | 1.000 |  |  |  | HBA | 0.124 | 0.257 | 0.283 | 1.000 |  |  |  |
| SBP | 0.246 | 0.189 | 0.149 | 0.093 | 1.000 |  |  | SBP | 0.215 | 0.135 | 0.151 | 0.126 | 1.000 |  |  |
| CHOL | 0.029 | 0.008 | 0.013 | 0.038 | 0.091 | 1.000 |  | CHOL | 0.025 | 0.027 | 0.018 | 0.032 | 0.071 | 1.000 |  |
| HDL | -0.304 | -0.109 | -0.143 | -0.127 | -0.042 | 0.176 | 1.000 | HDL | -0.271 | -0.090 | -0.193 | -0.079 | -0.026 | 0.144 | 1.000 |

Table vii: Correlation matrix Phase 9

|  | Real Whitehall Data: Phase 9 | | | | | | |  | Simulated Data: Phase 9 | | | | | | |
| --- | --- | --- | --- | --- | --- | --- | --- | --- | --- | --- | --- | --- | --- | --- | --- |
|  | BMI | G2HR | FPG | HBA | SBP | CHOL | HDL |  | BMI | G2HR | FPG | HBA | SBP | CHOL | HDL |
| BMI | 1.000 |  |  |  |  |  |  | BMI | 1.000 |  |  |  |  |  |  |
| G2HR | 0.152 | 1.000 |  |  |  |  |  | G2HR | 0.148 | 1.000 |  |  |  |  |  |
| FPG | 0.169 | 0.439 | 1.000 |  |  |  |  | FPG | 0.173 | 0.561 | 1.000 |  |  |  |  |
| HBA | 0.150 | 0.319 | 0.432 | 1.000 |  |  |  | HBA | 0.145 | 0.481 | 0.543 | 1.000 |  |  |  |
| SBP | 0.188 | 0.172 | 0.120 | 0.013 | 1.000 |  |  | SBP | 0.216 | 0.136 | 0.144 | 0.129 | 1.000 |  |  |
| CHOL | -0.003 | -0.007 | -0.018 | 0.007 | 0.122 | 1.000 |  | CHOL | 0.006 | 0.012 | 0.006 | 0.020 | 0.107 | 1.000 |  |
| HDL | -0.288 | -0.152 | -0.111 | -0.075 | -0.016 | 0.219 | 1.000 | HDL | -0.253 | -0.084 | -0.175 | -0.075 | 0.033 | 0.217 | 1.000 |
